# Electrophysiological and Molecular Mechanisms of Sinoatrial Node Mechanosensitivity

**DOI:** 10.3389/fcvm.2021.662410

**Published:** 2021-08-09

**Authors:** Daniel Turner, Chen Kang, Pietro Mesirca, Juan Hong, Matteo E. Mangoni, Alexey V. Glukhov, Rajan Sah

**Affiliations:** ^1^Department of Medicine, University of Wisconsin-Madison School of Medicine and Public Health, Madison, WI, United States; ^2^Cardiovascular Division, Department of Internal Medicine, Washington University School of Medicine, St. Louis, MO, United States; ^3^Institut de Génomique Fonctionnelle, Université de Montpellier, CNRS, INSERM, Montpellier, France

**Keywords:** automaticity, ion channel, cardiac, stretch activated, calcium, heart rate

## Abstract

The understanding of the electrophysiological mechanisms that underlie mechanosensitivity of the sinoatrial node (SAN), the primary pacemaker of the heart, has been evolving over the past century. The heart is constantly exposed to a dynamic mechanical environment; as such, the SAN has numerous canonical and emerging mechanosensitive ion channels and signaling pathways that govern its ability to respond to both fast (within second or on beat-to-beat manner) and slow (minutes) timescales. This review summarizes the effects of mechanical loading on the SAN activity and reviews putative candidates, including fast mechanoactivated channels (Piezo, TREK, and BK) and slow mechanoresponsive ion channels [including volume-regulated chloride channels and transient receptor potential (TRP)], as well as the components of mechanochemical signal transduction, which may contribute to SAN mechanosensitivity. Furthermore, we examine the structural foundation for both mechano-electrical and mechanochemical signal transduction and discuss the role of specialized membrane nanodomains, namely, caveolae, in mechanical regulation of both membrane and calcium clock components of the so-called coupled-clock pacemaker system responsible for SAN automaticity. Finally, we emphasize how these mechanically activated changes contribute to the pathophysiology of SAN dysfunction and discuss controversial areas necessitating future investigations. Though the exact mechanisms of SAN mechanosensitivity are currently unknown, identification of such components, their impact into SAN pacemaking, and pathological remodeling may provide new therapeutic targets for the treatment of SAN dysfunction and associated rhythm abnormalities.

## Introduction

**Figure 1:**
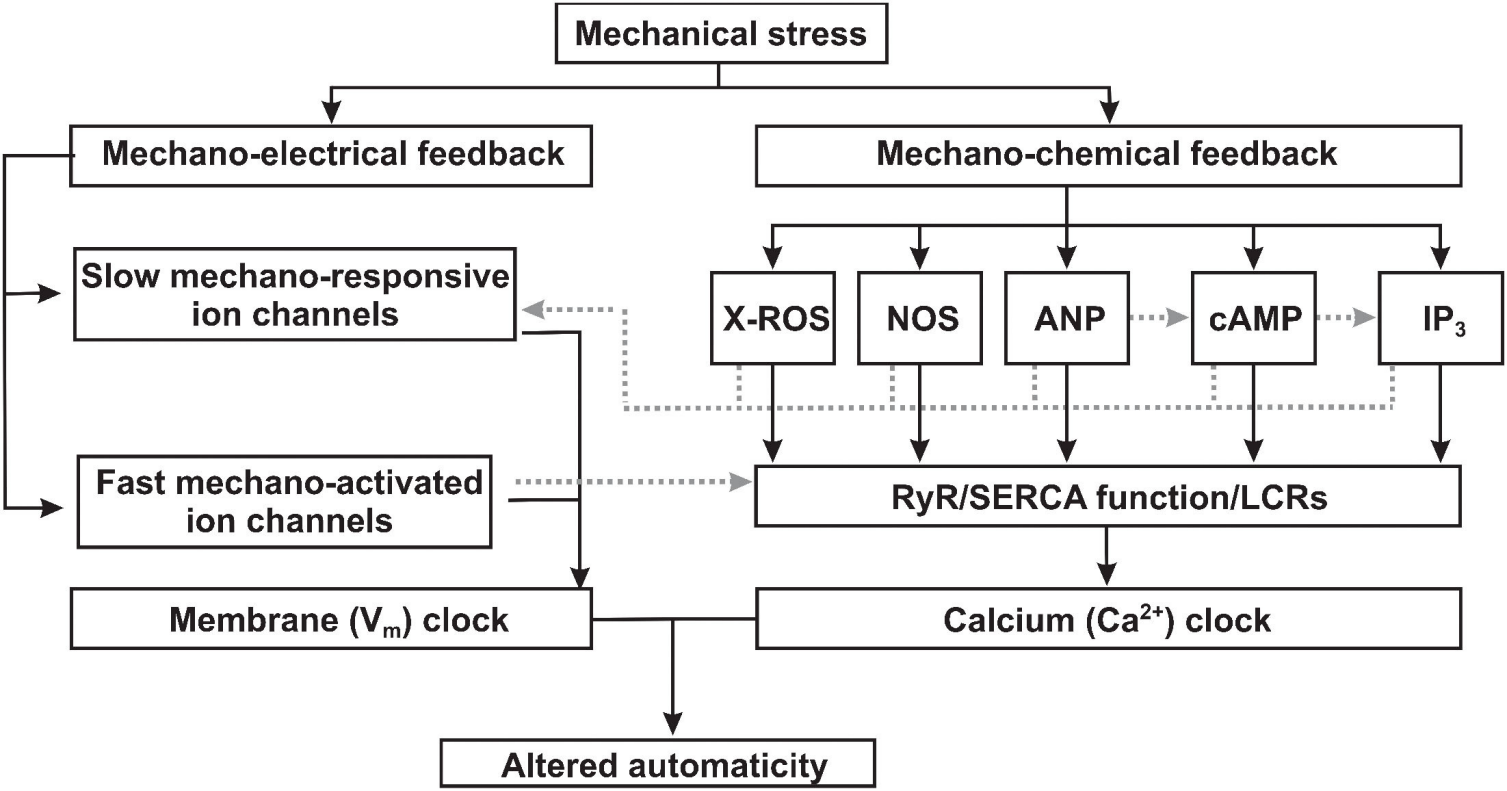
Proposed mechanisms of mechano-electrical and mechano-chemical feedback contributions to sinoatrial node mechanosensitivity. Mechanical stress (1) triggers mechano-electrical signal transduction pathways via both slow mechano-responsive and fast mechano-activated ion channels directly changing the membrane (V_m_) clock component of the coupled-clock pacemaker system; and (2) activates mechano-chemical feedback via various signaling factors which alters the function of the calcium (Ca^2+^) clock component of the coupled-clock pacemaker system. ROS, reactive oxygen species; NOS, nitric oxide synthase; ANP, atrial natriuretic peptide; cAMP, cyclic adenoside monophosphate; IP_3_, inositol triphosphate; RyR, ryanodine receptor; SERCA, sarcoplasmic reticulum Ca^2+^-ATPase; LCRs, local calcium releases.

**Figure 2:**
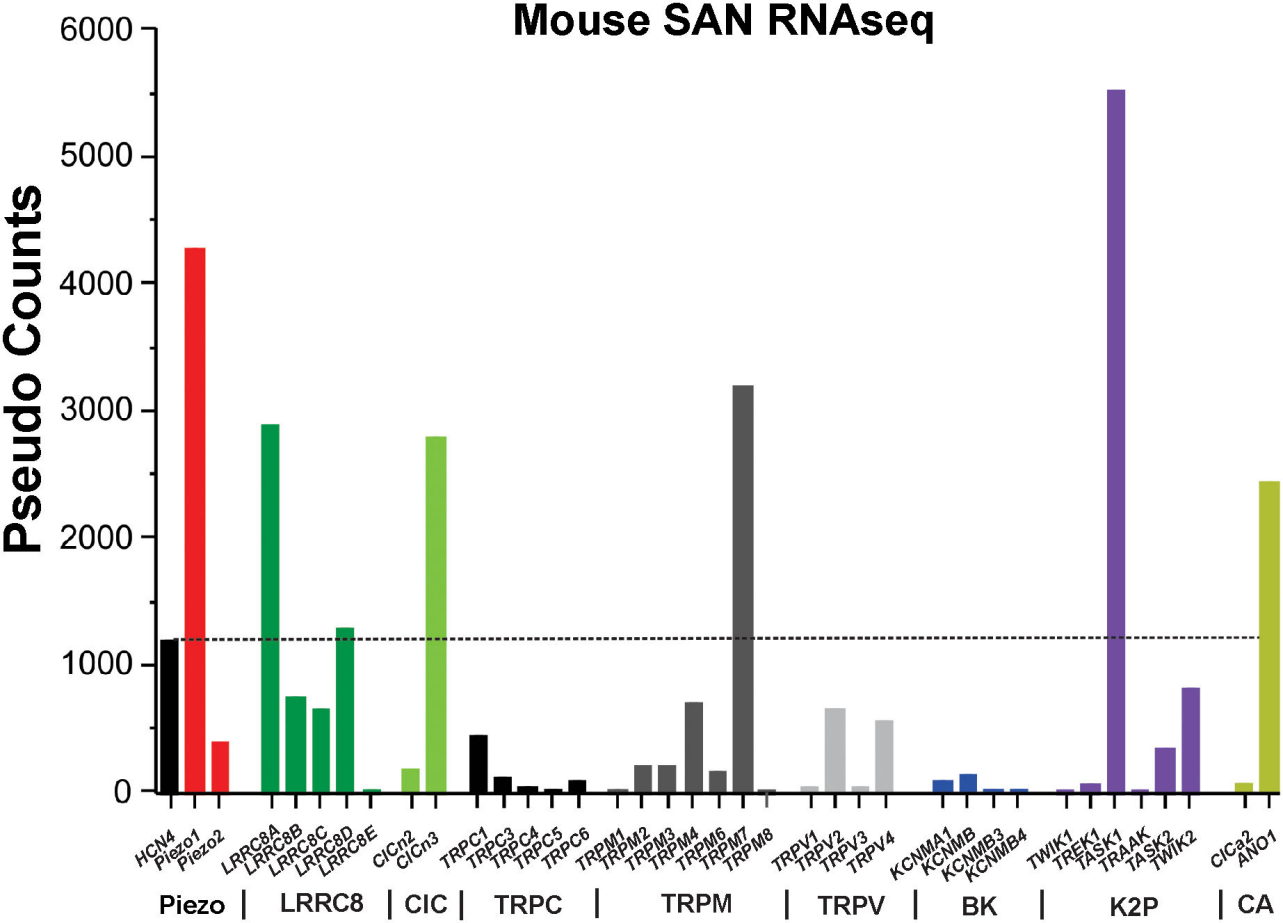
RNAseq of mouse sinoatrial node. The graph shows the absolute values (in pseudo-counts) for mRNA expression level. Horizontal dotted line indicates HCN4 level. LRRC8, leucine-rich repeat containing 8 family chloride channels; ClC, chloride channel; TRPC/M/V, transient receptor potential cation/melastatin/vanilloid subtype ion channels; BK, Ca^2+^-activated “big” potassium ion channels; K2P, two-pore domain potassium ion channels; CA, calcium-activated chloride ion channels; HCN4, hyperpolarization activated cyclic nucleotide gated cation channel 4.

The heart is continuously experiencing a dynamic mechanical environment, both on a beat-to-beat basis (e.g., fluctuating blood pressure and exercise) and chronically (e.g., elevated venous return and high blood pressure). Alterations in intra-cardiac pressure and/or volume preload/afterload may influence cardiac performance to coordinate cardiac output with venous return and arterial blood supply, in a cardiac autonomous fashion. This process involves activation of complex mechano-electrical [i.e., mechanically induced changes in cardiac action potential (AP) morphology, frequency, and propagation] and mechanochemical (i.e., changes in various second messenger signaling that are ultimately translated into regulation of calcium handling) signal transduction feedback mechanisms that autoregulate the frequency and the force of cardiac muscle contraction (Figure 1). An important component of such autoregulation includes changes in heart rate controlled by the heart's primary pacemaker, the sinoatrial node (SAN). SAN response to altered hemodynamic load is described via the Bainbridge response: an increase in heart rate upon right atrial pressure/volume increase, which may help in matching cardiac output to venous return (1). SAN mechanosensitivity and associated changes in pacemaker activity have been demonstrated at multiple levels, including isolated heart (2, 3) as well as in isolated SAN cells (4), and have been linked to mechanosensitive mechanisms (5, 6) intrinsic to pacemaker cells (7). In this review, we summarize the emerging understanding of cellular and molecular mechanisms that could be involved in SAN mechanosensing and pacemaker rate attenuation. Though the exact components of mechano-electro-chemical signal transduction, specifically involved in SAN mechanosensitivity, are not currently identified, here, we overview possible candidates that might be responsible for both fast (i.e., within seconds or on beat-to-beat manner) and slow (minutes) changes in SAN automaticity in response to mechanical stress. Specifically, we focus on canonical mechanoactivated channels (Piezo, TREK, and BK), slow mechanoresponsive ion channels (including volume-regulated chloride channels (ClC), and transient receptor potential (TRP) channels), and the components of mechanochemical signal transduction, including reactive oxygen species (ROS), cyclic adenosine monophosphate (cAMP), and inositol trisphosphate (IP_3_). Mechanosensitivity of these pathways has been described in either non-pacemaker cardiomyocytes (atrial or ventricular) or non-cardiac cells. Here, we show the expression profile of mechanosensitive ion channels in murine SAN (Figure 2) and discuss how these ion channels, as well as various mechano-chemical signaling pathways, could potentially modulate membrane and calcium clock components of the so-called coupled-clock pacemaker system (8), contributing to SAN mechanosensitivity and changing in heart rate upon alterations in intra-cardiac mechanics.

## Sinoatrial Node Anatomy

The SAN is a small body of specialized cardiac tissues located within the wall of the right atrium of the heart, laterally to the entrance of the superior vena cava, anatomically described by Silverman and Hollman (9). The SAN has a crescent-shaped structure positioned along the crista terminalis and running between the superior and inferior venae cavae, usually being arranged around a prominent nodal artery. The SAN is functionally insulated from the surrounding atrial myocardium, except for several critical conduction pathways (10–13). Indeed, the SAN requires anatomical (fibroblasts, adipose tissue, and blood vessels) and/or functional barriers (paucity of connexins) (13–16) to protect it from the hyperpolarizing influence of the surrounding atrium in order to function as a leading pacemaker. The presence of conduction barriers and pathways may explain how a small cluster of pacemaker cells in the SAN pacemaker complex manages to depolarize separate, widely distributed areas of the right atrium as evidenced functionally by exit points and breakthroughs (17–21). The autonomic nervous system and humoral factors can further regulate conduction through these pathways, contributing to pacemaker automaticity and ultimately determining heart rate (22–24).

## Mechanosensitivity of the Sinoatrial Node

The SAN is well-positioned anatomically to sense both coronary and atrial blood pressure changes, providing a structural basis for hemodynamic regulation of heart rate via SAN mechanosensitivity. Changes in venous blood flow to the heart not only affect the volume available for atrial contraction and subsequent ventricular filling but also has an impact on the diastolic atrial dimension. Increased right atrial filling distends the atrial wall, including the SAN myocytes, which may consequently influence the pacemaker function and heart rate. This mechano-modulation of pacemaker activity was first described in 1915, when Bainbridge observed an increase in heart rate associated with right atrial distension from intravenous fluid injection in anesthetized dogs (1). While Bainbridge originally attributed this phenomenon to altering autonomic inputs, a study performed in dogs by Brooks et al. (25) determined that this positive chronotropic response was insensitive to adrenergic and cholinergic receptor blockade, and also to denervation, suggesting SAN intrinsic regulation of SAN automaticity. Conversely, in 1963, James and Nadeau demonstrated a bradycardic SAN response in dogs upon injection of fluid into the right atrium, while controlling for temperature, pH, osmolarity, oxygen, and ionic content (3). It was not until 1978, when Donald and Shepherd (2) performed controlled observations of human atrial and SAN mechanosensitivity by developing an experimental method that did not increase arterial blood pressure in humans (baroreceptor “depressor reflex”), that SAN mechanosensitivity was observed in humans. By placing subjects into a “supine” position, these researchers were able to observe an increase in heart rate concurrent with an increase in venous return to the heart. Lastly, Cooper et al. (4) determined that direct moderate stretch on isolated SAN cells, a possible consequence of increased venous pressure *in vivo*, induced elevated beating rate, confirming “for the first time, that the positive chronotropic response of the heart to stretch is, at least in part, encoded on the level of individual sinoatrial node pacemaker cells.” Please refer to the review by Quinn and Kohl for a deeper examination on the history of SAN mechanosensitivity and canonical mediators (6).

While these cornerstone studies demonstrate the immediate or “fast” response of stretch on SAN automaticity, there is also growing evidence of “slow” activating channels (>1 min), ClC-2, for example (26), which can be activated by long-term pressure increases normally associated with the slow force response in the working myocardium (27). Prior to its naming, the slow force response was observed in feline and canine models. Gertrude et al. observed in isolated cat nodal tissue that sustained stretch accelerated beating rate and even induced spontaneous beating from quiescent nodal cells (28). From a similar group of researchers, Brooks et al. observed a similar response in anesthetized dogs (25). Using *in situ* SAN stretch, they observed a biphasic response to SAN stretch with an immediate acceleration of beating rate followed by a decrease to a rate still above pre-stretch levels (25). These gradual (over the course of minutes) and reversible changes in beating rate and cardiac contractility inherent of the slow force response may play a role in more delayed changes in SAN automaticity via slowly activating mechanosensitive channels (26, 29, 30) and various mechano-chemical signaling pathways (31–35).

## Electrophysiological Mechanisms of Sinoatrial Node Mechanosensitivity

### Overview of Sinoatrial Node Pacemaker Activity

**Figure 3:**
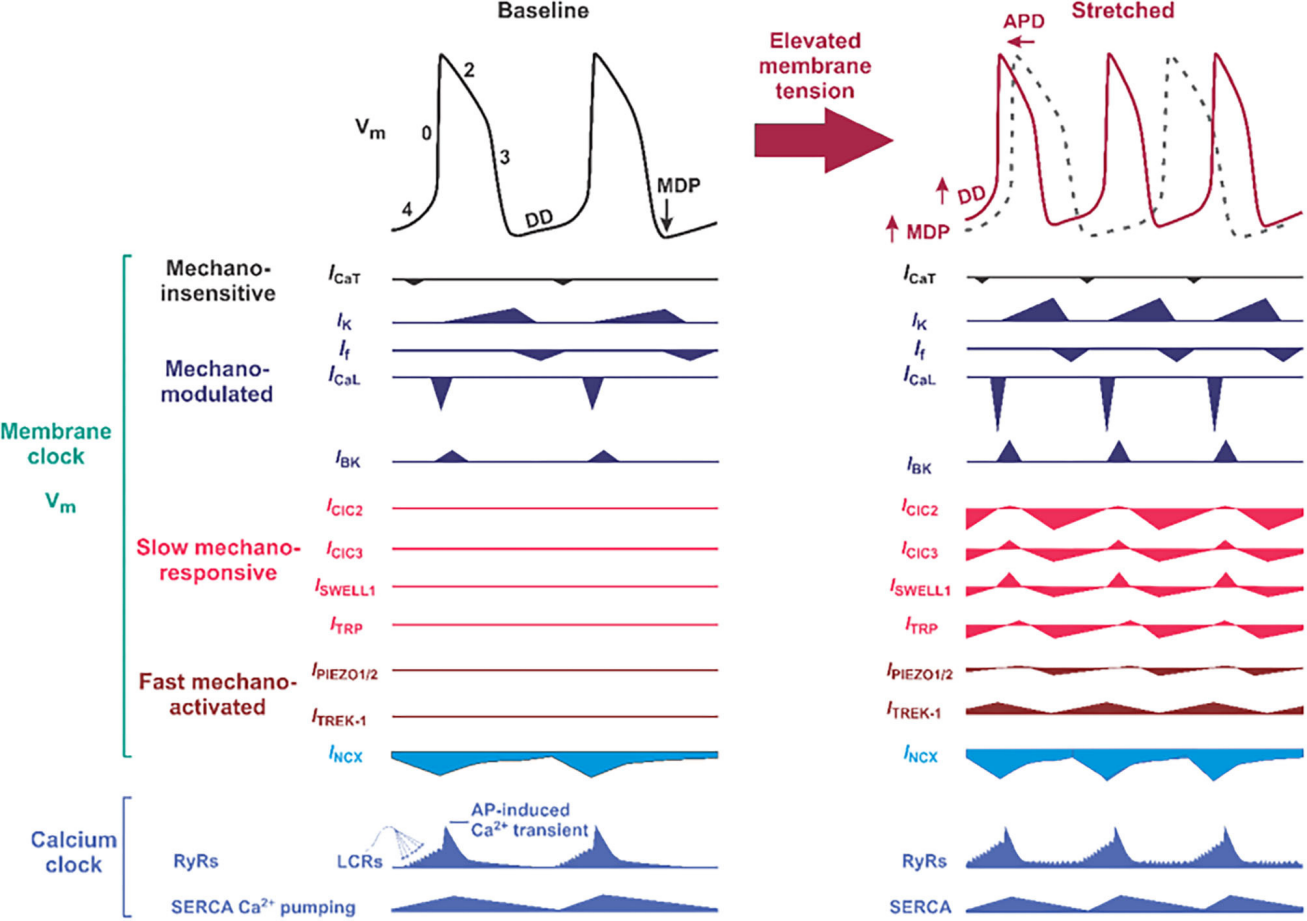
Proposed molecular composition of the mechano-electrical signal transduction in the sinoatrial node (SAN) cell. (**Left**) Typical SAN membrane action potential (black trace) and the timing of membrane (V_m_) clock and calcium (Ca^2+^) clock components of the coupled-clock pacemaker system are shown. The phases of the action potentials are labeled including phase 4, in which diastolic depolarization (DD) that is key to automatic pacemaker activity takes place. APD, action potential duration; MDP, maximum diastolic potential; DD, diastolic depolarization; *I*_Ca,T_ and *I*_Ca,L_, T- and L-type voltage-dependent Ca^2+^ currents; *I*_NCX_, sodium-calcium exchange current; *I*_K_, rapid (*I*_Kr_) and slow (*I*_Ks_) delayed rectifier potassium currents; *I*_f_, HCN4 “funny” current; SERCA, sarco-endoplasmic reticulum ATPase; LCRs, local Ca^2+^ releases. Below “classical” ion channels defined as mechano-modulated as indicated by various authors (38–40) which can have their normal activity altered by mechanical stress, proposed slow mechano-responsive, and fast mechano-activated ion channels are shown. ClC, chloride channels; SWELL1, swelling-activated leucine-rich repeat containing 8 (LRRC8) family chloride channels; TRP, transient receptor potential ion channels. (**Right**) Proposed changes in SAN action potential morphology (solod red trace on top of the black dotted trace for baseline condition) under mechanical stress. Below, proposed contribution of slow mechano-responsive and fast mechano-activated ion channels is shown for each ion channel.

Spontaneous beating of SAN myocytes is initiated, sustained, and regulated by a complex coupled system of cellular “clocks” that integrates ion channels and transporters on the cell membrane surface or “voltage clock,” with subcellular Ca^2+^ handling machinery, also referred to as an intracellular “Ca^2+^ clock” (8, 36, 37) (Figure 3). The firing of SAN cells is due to diastolic depolarization, a slow depolarizing phase of the membrane potential (V_m_), mediated by the concomitant action of both membrane and Ca^2+^ clocks. Since SAN cells lack *I*_K1_ current expression (41), following the minimum, or most hyperpolarized diastolic potential, potassium *I*_K_ current (*I*_Ks_, and *I*_Kr_) conductance decreases, allowing the inward hyperpolarization-activated current (*I*_f_) (42, 43) and a low-threshold, voltage-gated T-type Ca^2+^ current (*I*_Ca,T_), which contribute to the early fraction of diastolic depolarization (Figure 3) (44). In addition, L-type Ca_v_1.3 Ca^2+^ channels open during diastolic depolarization to generate an inward Ca^2+^ current (45, 46) and enabling the sustained inward Na^+^ current *I*_*st*_ (47). Local Ca^2+^ release (LCR) from the sarcoplasmic reticulum (SR) via subsarcolemmal ryanodine receptors (RyRs) generates small increments in intracellular Ca^2+^ concentration. These LCRs activate Na^+^/Ca^2+^ exchange (NCX) to pump Ca^2+^ out of the cell in exchange for Na^+^ ions at a ratio of 1 Ca^2+^:3 Na^+^, to generate an inward NCX current (*I*_NCX_), and this contributes to both early and late phases of diastolic depolarization (48) and subsequent depolarization of the V_m_ to the threshold of the next beat (Figure 3). The exact molecular mechanisms responsible for SR Ca^2+^ release during late diastole are not completely understood. While some studies show that such local Ca^2+^ release events are spontaneous, independent of transmembrane potential, and likely include stochastic opening of hyperphosphorylated RyRs (49–51), other evidence suggest that these events might be triggered by Ca^2+^ entry via low-voltage activated T-type Ca^2+^ channels (52) or Ca_v_1.3 L-type Ca^2+^ channels (46). Particularly, recent studies indicate that Ca_v_1.3 channel activity contributes to generation and synchronization of diastolic LCRs (46) and that Ca_v_1.3 is necessary for the Ca^2+^ clock function during SAN firing (53). Overall, the sum of *I*_f_, *I*_Ca,T_, *I*_NCX_ and Ca_v_1.3-mediated L-type Ca^2+^ current (*I*_Ca,L_) contributes to diastolic depolarization required to ultimately trigger activation of cardiac Ca_v_1.2-mediated *I*_Ca,L_ that initiates the AP, and global Ca^2+^-induced Ca^2+^ release. In nature, neither clock functions in the absence of the other. Abundant evidence indicates that functional interactions between the two clock components are critical for normal SAN automaticity (8, 36, 37, 46).

There has been significant interest in determining the underlying cellular and molecular mechanisms responsible for intrinsic SAN mechanosensitivity. Mechanical modulation of SAN pacemaking adds another level of complexity to SAN automaticity that has been proposed by Quinn and Kohl as the additional “mechanics-clock” (6, 7) or, more accurately, as a third coupled oscillator. The authors specifically highlighted that in case of fast, beat-to-beat changes in heart rate, the voltage and Ca^2+^ clocks do not inherently account for the rapid response of the SAN to changes in hemodynamic load and that another set of mechanisms must contribute to spontaneous diastolic depolarization of the SAN. The importance of “mechanics-clock” could be further supported by the fact that stretching of quiescent tissue frequently induces spontaneous activity. In particular, arrhythmic isolated hearts of *Prosobranch* gastropod become rhythmic when the pacemaker tissue is stretched by with internal perfusion and improve in form as pressure is increased (25). It may be more accurate to describe mechanical modulation of SAN pacemaking as an additional coupled oscillator since it could be applied to both fast and slow changes in heart rate through coupling with fast and slow mechanical oscillators, respectively.

Since ion channels are both central for the regulation of SAN automaticity and can sense mechanical stimuli via various mechanisms, they provide plausible molecular candidates for SAN mechanosensitivity. Ion channels may be grouped into two categories with respect to mechanosensitivity: (1) directly mechanoactivated (fast) and (2) indirectly mechanoresponsive (slow) (Figure 1). Although both categories of channels can change their open probability and other biophysical characteristics in response to mechanical stimulation, they differ in how mechanical forces transduce these effects. Fast directly *mechanoactivated* ion channels (Piezo1-2, TREK-1, TRAAK, and BK channels) are intrinsically sensitive to mechanical forces applied to the protein or to the lipid bilayer in which the channel resides and do not require any other associated proteins or protein complexes to confer mechano-responsiveness (54, 55). Slow indirectly *mechanoresponsive* ion channels are polymodal ion channels (TRP channels, SWELL1/LRRC8, and ClC) that respond to mechanical forces in cell-type specific contexts but may not themselves be intrinsically mechanosensitive, for example, when reconstituted in a minimal lipid membrane, devoid of other cellular proteins, with some channels displaying faster activation kinetics and some slower.

## RNA Sequencing Identifies Highly Expressed and Enriched Mechanosensitive Ion Channels in Mouse Sinoatrial Node

There are a multitude of mechanosensitive and mechanoresponsive ion channels expressed in mammalian cells. However, without knowledge of the expression level of these channels in SAN, their relevance to SAN physiology is entirely speculative. To guide our discussion of the molecular mechanisms of SAN mechanosensitivity, we examined the expression levels of Piezo1-2, LRRC8a-e, TRPCs, TRPVs, TRPMs, K2P, and ClCs in a genome-wide RNA sequencing data set derived from murine SAN (Figure 2). Hyperpolarization-activated cyclic nucleotide-gated potassium channel 4 (HCN4) is robustly expressed and enriched in murine SAN. Remarkably, the most highly expressed mechanosensitive and mechanoresponsive ion channels (or essential components) in SAN are Piezo1 (3.6-fold greater than HCN4), LRRC8a [SWELL1/volume-regulated anion channel (VRAC), 2.4-fold greater than HCN4], ANO1 (2.04-fold greater than HCN4), and TASK-1 (4.6-fold greater than HCN4). Among TRP channels, the cells expressing TRPM7 were the most present (2.2-folds more than cells expressing HCN4), followed by cells expressing TRPM4, TRPV2, TRPV4, and TRPC1. Regarding mechanosensitive K2P channels, the number of counts for TREK-1 was lower than the number of HCN4. RNA sequencing data did not detect any cells expressing TRAAK. On the contrary, there were more counts for TASK1 than HCN4. Among ClC channels, the number of CICn3 counts was more than that for HCN4, the opposite for CICn2. Furthermore, RNA sequencing showed that the BK channel alpha subunit (KNCMA1) is lowly represented in SAN. Based on these data, we will discuss primarily those mechanosensitive/responsive ion channels that are highly expressed in SAN relative to HCN4. However, one limitation of mouse SAN is that they are the only known species to have a negative chronotropic response to sustained SAN stretch (56). Furthermore, please refer to Table 1 as a resource for where the following ion channels have been confirmed, how they were analyzed, and their literature sources.

**Table 1:** Compiled mechanosensitive ion channels discussed in the review, their known expression and detection method, and respective references.

**Channel**	**Expression**	**References**
Piezo1	Mouse heart homogenate (PCR), Mouse SAN (RNAseq)	(57); Present Publication
Piezo2	Mouse SAN (RNAseq)	Present Publication
LRRC8a	Mouse heart homogenate (PCR), rat and human atria (PCR, WB, IHC, IP), Mouse SAN (RNAseq)	(58, 59) Present Publication
LRRC8b-e	Mouse SAN (RNAseq)	Present Publication
ClC-2	Rat SAN (PCR), guinea pig SAN (PCR, IHC, ICC), mouse SAN (PCR, KO)	(26, 60, 61)
ClC-3	Mouse SAN (PCR), mouse atria (PCR, WB, KO), rat and human atria (PCR, WB, IHC, IP)	(59, 60, 62)
TRPC6	Rat SAN (PCR), mouse SAN (PCR, ICC)	(61, 63)
TRPM4	Mouse SAN (PCR, WB), mouse SAN (KO)	(64, 65)
TRPM7	Mouse SAN (PCR, WB, IHC, ICC, KO)	(66, 67)
TRPV1-4TREK-1	Mouse SAN (RNAseq) Rat and human SAN (PCR), mouse SAN (PCR, WB, IHC, KO), rabbit SAN (WB)	Present Publication (61, 68, 69)
TASK-1	Rat and human SAN (PCR)	(61, 68)
TASK-2	Mouse, rat, and human SAN (PCR)	(60, 68)
BKClCa_2_	Mouse SAN (WB PCR, and IF) Mouse SAN (IF and RNA seq)	(70) Present Publication (71)
ANO1	Mouse SAN (RNAseq)	Present Publication

## Directly Mechanoactivated Ion Channels

### Piezo Channels

Piezo1 and Piezo2 ion channels are bona fide mechanoactivated cation channels with established roles in the cardiovascular system (57, 72) and provide another potential mediator of SAN mechanosensitivity. These non-selective cationic channels may be activated by shear stress from nearby blood flow (laminar or turbulent) as well as membrane stretch induced by increased blood pressure, and their activation is highly sensitive to mechanical stimulus variation in frequency and duration (73). In addition, they are non-selective and are therefore permeable to Ca^2+^ and Na^+^, in addition to K^+^, and have a relatively low threshold for mechanoactivation (57, 74). Although there have been no studies directly examining Piezo1 in the SAN, it is expressed in cardiac tissue (54) and appears highly expressed in the murine SAN (Figure 2). Moreover, Piezo1 plays an important role in the regulation of vascular tone (54) and baroreceptor pressure sensing (55). Since Piezo1 channels provide a depolarizing current in response to mechanoactivation, they are good candidates for mechanically activated increases in SAN automaticity and heart rate acceleration (72). Moreover, Piezo1 channels activate rapidly (within milliseconds) and are responsive to phasic, high-frequency mechanical inputs, such as systolic contractions, but may also be modulated by more gradual mechanical inputs (75), such as increases in atrial filling pressures and could therefore govern SAN mechanosensitivity on a beat-to-beat basis and during periods of chronic stretch. Future studies with targeted genetic deletion of Piezo1 from SAN may directly test these hypotheses.

### TREK-1 Channels

Cardiac cells have two-pore domain potassium currents with little time- or voltage-dependency, also known as background currents, that regulate resting membrane potential and cell excitability (76). The family of cloned mammalian background K^+^ channels includes 14 members encoded by different genes. The members were divided into six subfamilies, TWIK, TREK, TASK, TALK, THIK, and TRESK, on the basis of sequence homology and functional similarities (77). Two-pore domain potassium channels are typically insensitive to conventional K^+^ channel blockers such as 4-AP, TEA, Ba^2+^, Cs^+^, and glibenclamide (76), but they are sensitive to membrane stretch, changes in extracellular or intracellular pH, fatty acids, and inhalation anesthetic agents (e.g., isoflurane) and are regulated by second messenger phosphorylation (76). Structurally, these channels possess four transmembrane domains and two pore domains; each subunit contains two pore-forming domains, so two subunits can form a complete pore of the channels. In murine SAN (Figure 2), two subtypes of two-pore domain potassium channels are mainly expressed: stretch-activated K^+^ channel TREK-1 (78–80) and the acid-sensitive K^+^ channel TASK1 (81–83). Under basal conditions, the activity of the TREK channels is low; however, applying negative pressure to the cell membrane reversibly activates TREK-1 (84). In addition, laminar shear stress stimulates TREK-1, whereas the cell shrinkage induced by extracellular hyperosmolarity reduces the amplitude of TREK-1 (84). Indeed, it has been shown that TREK-1 mechanosensitivity is mediated directly by the lipid membrane perturbations and changes in plasma membrane tension (85). Given its expression in the SAN (Figure 2), it is a candidate contributor to SAN mechanosensitivity on a fast (<1 s) basis.

A number of studies on zebrafish and mice that inhibited plasma membrane trafficking of TREK-1 by inactivating the interacting proteins POPDC1 and POPDC2 revealed exercise- and age-dependent sick sinus syndrome and atrioventricular block (78, 86), suggesting a role for TREK-1 in cardiac automaticity. Similarly, transgenic overexpression of a C-terminal truncation of beta IV spectrin, which also disrupts TREK-1 plasma membrane trafficking, results in sick sinus syndrome (87). These studies provide indirect evidence of TREK-1-mediated effects on SAN automaticity. However, more direct evidence was provided by Unudurthi et al. (69). The authors determined that TREK-1 protein is indeed expressed in both murine and rabbit SAN, and TREK-1-like background currents were reduced in patch-clamped SAN cells isolated from cardiac-specific TREK-1 KO mice (αMHC-*Kcnk2*^f/f^). Also, freely moving, telemetered αMHC-*Kcnk2*^f/f^ mice exhibited sinus bradycardia at rest, consistent with studies by Hund et al. (87) where disrupted plasma membrane TREK-1 trafficking induced sick sinus syndrome. Paradoxically, isolated TREK-1 KO SAN cells exhibited increased rather than decreased firing rates as compared with wild-type (WT) SAN. Furthermore, exercise and treatment with epinephrine uncovered stress-induced sinus pauses in αMHC-*Kcnk2*^f/f^ mice via unclear mechanisms, or possibly via variation in sympathetic and parasympathetic activity. Finally, intrinsic heart rates measured in telemetered αMHC-*Kcnk2*^f/f^ mice with atropine and propranolol treatment exhibited no significant differences, suggesting that neurohumoral inputs are important for TREK-1-dependent regulation of SAN automaticity. These studies illustrate the incomplete understanding of TREK-1 and its contribution to SAN mechanosensitivity, which requires further investigation.

### BK Channels

BK (large-conductance Ca^2+^- and voltage-activated K^+^) channels are another promising contributor to SAN mechanosensitivity and automaticity [reviewed in (88)]. These channels are characterized as having large single-channel conductance and selective inhibitors and are regulated by voltage and Ca^2+^ (70). Imlach et al. (89) determined that BK channel inhibition via paxilline caused a reduction in heart rate in isolated mouse and rat hearts but not in hearts from *Kcnma1* KO mice. This finding was confirmed at a cellular level when Lai et al. (70) demonstrated that paxilline applied directly to isolated mouse SAN cells reduced AP firing rate in WT mice but not in *Kcnma1* KO mice. Lastly, Zhao et al. (90) found that BK channels are mechanosensitive to a small degree, showing an increased activity in chick ventricular myocytes plated on stretched extracellular matrix. Given this and their expression in murine SAN (Figure 2), BK channels are a putative contributor to SAN mechanosensitivity. In this case, SAN membrane depolarization, increases in cytosolic Ca^2+^, and mechanical stimulation from SAN/atrial systole all coincide to activate BK channels after the peak of the SAN AP to augment AP repolarization, re-initiation of diastolic depolarization, and heart rate acceleration (Figure 3). Based on this model, mechanoactivation of BK channels must be relatively rapid to contribute to SAN AP repolarization, as published data suggest; however, it remains unclear if these channels are rapidly or slowly mechanoactivated in SAN.

### Mechanoresponsive Transient Receptor Channels

Putative candidates for stretch-responsive non-selective cation channels include TRP channels expressed in murine SAN: TRPC1, TRPC3, TRPM4, TRPM7, TRPV2, and TRPV4 (Figure 2). A number of TRP channels that we found expressed in murine SAN have been described as mechanoresponsive either directly or indirectly (29, 91). However, thus far, only TRPM4 and TRPM7 have been studied in the context of SAN function. TRPM4 is an intracellular Ca^2+^-activated, non-selective cation channel, which is possibly indirectly mechanoresponsive (29). At negative membrane potentials, TRPM4 activation allows Na^+^ influx, leading to the membrane depolarization, whereas, at the positive membrane potentials, TRPM4 allows K^+^ efflux, leading to membrane repolarization (92, 93) (Figure 3). In rodent SAN, TRPM4 is thought to contribute to diastolic depolarization and a positive chronotropic response in response to stretch (64, 65, 94). TRPM7, an ion channel and protein kinase (chanzyme), permeable to both divalent cations, including Zn^2+^, Mg^2+^, and Ca^2+^, as well as monovalent cations such as Na^+^ and K^+^ (95, 96), is broadly expressed. TRPM7 is highly expressed in murine SAN at the mRNA level (Figure 2) and generates a robust current in both SAN and atrioventricular node cells (66). Both cardiac- and SAN-targeted TRPM7 deletion impaired cardiac automaticity (67); however, the mechanism was proposed to be via regulation of *HCN4* and *I*_f_ current rather than a direct effect on diastolic depolarization via channel activity (66, 67). While it is clear that none of these TRP channels are intrinsically mechanoactivated (97, 98), it is possible that some of these channels form part of a mechanosensory system (29) and therefore may be mechanoresponsive within specific cellular contexts (99). Testing these hypotheses would require directly measuring these mechanoresponsive currents in isolated SAN, as performed by Kohl et al. (4, 100), in genetic knock-outs of each of these putative mechanoresponsive channels or using specific pharmacologic inhibitors.

### Volume-Regulated Anion Channels

Another mechanoresponsive ionic current that has been implicated in the regulation of SAN automaticity is *I*_Cl,SWELL_ or the swell-activated chloride current. This ionic current may be carried by VRAC or ClC ion channels, both of which are most commonly activated by cell swelling, which is typically achieved by applying hypotonic or hypo-osmolar solution to cells. However, in a few studies, anion or chloride conductances were demonstrated by application of mechanical forces, as described in further details below. VRACs are activated by cell swelling, ubiquitously expressed in various mammalian cell types and thought to be implicated in many physiological and pathophysiological processes, including fluid secretion, glutamate release, membrane potential regulation, and apoptosis [summarized in the review article (101)]. Although the biophysical properties of VRACs have been well-characterized in multiple cell types over the course of decades, the molecular identity of VRAC remained a mystery until the Patapoutian (58) and Jentsch (102) groups identified leucine-rich repeat containing 8a (LRRC8a, also known as SWELL1) as a required component of a heterohexameric channel complex consisting of various stoichiometries of LRRC8a, and/or LRRC8b,c,d,e. Although the function of the VRAC current has been attributed to cell volume regulation in response to relatively non-physiological osmotic gradients, the broad tissue expression pattern of LRRC8 proteins and presence of VRAC/*I*_Cl,SWELL_ current in a multitude of cell types (103–108), including cardiac myocytes (109–113) that are rarely exposed to hypotonic swelling, suggests that the actual physiological role of VRAC and LRRC8 channels remains unknown. Indeed, experiments using magnetic dynabeads bound to monoclonal antibodies for beta1-integrins demonstrated activation of *I*_Cl,SWELL_ in cardiac myocytes in response to mechanical force applied via magnetic fields (109, 110), supporting the notion that *I*_Cl,SWELL_ is mechanoresponsive in cardiac cells. Therefore, given the high mRNA counts of LRRC8a (SWELL1) and associated subunits LRRC8b,c,d in murine SAN relative to HCN4 (Figure 2), the contribution of SWELL1-mediated *I*_Cl,SWELL_ to pacemaker activity and response to stretch warrants further investigation.

Elegant studies by Hagiwara et al. (114) demonstrated that mechanical inflation of isolated rabbit SAN cells using positive pressure via the patch-pipette in whole-cell configuration induces an outwardly rectifying, stretch-activated anion current that is inhibited by chloride channel blockers, 4,4′-diisothiocyano-2,2′-stilbenedisulfonic acid (DIDs) and 9-anthracenecarboxylic acid (9-AC). Also, this current exhibits a shift in reversal potential consistent with a chloride conductance (115) and has a sequence of anion permeability (I^−^ > ${\text{NO}}_{3}^{-}$ > Br^−^ > Cl^−^ > F^−^) similar to VRAC or LRRC8 channels. *I*_Cl,SWELL_ activates over the course of minutes (~2 min), which implies responsiveness to tonic changes in membrane tension, as may be expected from gradual atrial stretch-associated increased venous return, but relatively unaffected by phasic changes associated with beat-to-beat changes. Based on the outwardly rectifying current–voltage relationship, and reversal potential around the Cl^−^ reversal potential (E_Cl_ = −30 mV), we speculate that inward chloride current at voltages below −30 mV may contribute to diastolic depolarization and SAN automaticity, while outward current at voltages above −30 mV may contribute to SAN AP shortening (116) (Figure 3). The integrated effects on automaticity and the response to stretch, however, are likely to be complex.

Similarly, Decher et al. found in guinea-pig atrial myocytes that *I*_Cl,SWELL_ induced by osmotic swelling leads to a shortening of AP duration that was inhibited by DCPIB (a relatively selective *I*_Cl,SWELL_ inhibitor) (117). Furthermore, Seol et al. found the *I*_Cl,SWELL_ can be activated by axial stretch in cardiomyocytes isolated from the pulmonary vein (30, 59); and Egorov et al. determined that *I*_Cl,SWELL_ activation in response to mechanical stretch can depolarize resting membrane potential, generate arrhythmic substrates, and confirm that it can also shorten APs (59). In isolated rabbit SAN tissue, Arai et al. also showed that application of various non-specific anion channel blockers that can block VRACs, such as DIDs, caused a reduction in the stretch-induced increase in firing rate at a high level of distension (118). On the other hand, Cooper et al. (56) reported that the application of 9-AC at 1 mM concentration had no effect on the stretch-induced increase in heart rate when a significant stretch-stimulus was applied, suggesting that *I*_Cl,SWELL_ may not underlie the SAN response to mechanical stretch. However, application of such high concentrations of 9-AC is highly non-specific and therefore complicates the interpretation of this result. Furthermore, the use of different stretching techniques between the two studies may account for the differences observed. These studies, albeit contradictory, indicate the potential role of *I*_Cl,SWELL_ in modulating SAN function on a slow, non-beat-to-beat basis, which may be present during periods of chronic stretch, and demonstrate the need for additional experiments. Since SWELL1 (LRRC8a) and LRRC8 subunit proteins are now known to encode *I*_Cl,SWELL_ in numerous other cell types (103–108), future studies examining cardiac specific and SAN targeted *Swell1* KO mice, transient knockdown in isolated cells, and/or more specific small molecules such as DCPIB will provide important new insights into the contribution of *I*_Cl,SWELL_ and VRAC in cardiac automaticity and the response to SAN stretch.

### ClC Anion Channels

Other candidates for the molecular identity for *I*_Cl,SWELL_ in SAN are the ClC ion channels. While both ClC-2 and ClC-3 have been studied in cardiac myocytes (26, 62), and ClC-3 has a high mRNA count in murine SAN (Figure 2), only ClC-2 has been directly studied with respect to regulating SAN automaticity. Huang et al. (26) showed that inwardly rectifying chloride current induced by osmotic swelling in isolated guinea-pig SAN pacemaker myocytes could be blocked though intracellular dialysis of anti-ClC-2 antibody, which did not affect other pacemaker currents, including *I*_f_, *I*_Ca,L_, and *I*_Ks_ and the volume-regulated outwardly rectifying Cl^−^ current (*I*_Cl,vol_). Anti-ClC-2 antibody reversed the changes in SAN APs induced by osmotic swelling. The authors also showed that ClC-2 KO (*ClCN2*^−/−^) mice demonstrate a decreased chronotropic response to acute exercise stress when compared with their age-matched *ClCN2*^+/+^ and *ClCN2*^+/−^ littermates. It was then concluded that targeted inactivation of ClC-2 does not alter intrinsic heart rate but prevented the positive chronotropic effect of acute exercise stress through sympathetic regulation of ClC-2 channels. While ClC-2 channels may contribute in part to cardiac *I*_Cl,SWELL_, there have been no studies examining the signaling mechanisms underlying ClC-2 mechanoresponsivity.

ClC-3, on the other hand, has been proposed to be mechanoresponsive in osteoblasts (119) and has been shown to be expressed in cardiac myocytes, to underlie *I*_Cl,SWELL_, and to be involved in numerous pathophysiological processes, including ischemic preconditioning, myocardial hypertrophy, and heart failure (120). However, there have yet to be any studies directly examining ClC-3 in SAN cells, and neither global nor cardiac specific ClC-3 KO mice were noted to show differences in heart rates (62).

### Other Chloride Channels

Other possible contributors to SAN mechanosensitivity are calcium-activated chloride channels (CaCCs) such as anoctamin1 (ANO1) and chloride channel accessory 2 (ClCA2). Ye et al. (121) determined that ANO1 is expressed in mouse ventricular myocytes and facilitates accelerated AP repolarization. Given that ANO1 is implicated to be mechanoresponsive (121) and expressed in murine SAN (Figure 2), it is plausible that ANO1 may contribute similarly to shorten pacemaker potentials, as Sung et al. speculated (122). In addition, Mao et al. found that ClCA2 is highly expressed in SAN tissue and, when mutated, induces mild conduction block and ectopic pacemaker activity (71). While no study has examined ClCA2 mechanosensitivity, given its calcium-activated properties, it is likely to be affect by pressure-induced calcium transients (123). Given these findings, it is feasible that calcium-activated chloride channels could play a partial role in the response of SAN beating rate to stretch.

## Caveolae-Mediated Ion Channel Mechanosensitivity

Interestingly, besides ANO1 and ClCA2, all the aforementioned ion channels affected by mechanical stress have been found to associate with caveolae, which are abundantly expressed in SAN cells (5, 124, 125) and are known to mediate cellular response to mechanical stress by reserving “extra” cell membrane to buffer mechanical forces and contribute to cell volume regulation (126–128). Caveolae are small, 50- to 100-nm omega-shaped membrane invaginations of the plasma membrane enriched by sphingolipids, cholesterol, cavin proteins, and caveolin proteins. Caveolin-3 (129) is the dominant isoform in muscle cell caveolae; however, caveolin-1 has also been found in atrial myocytes (129, 130). It has been shown that caveolae compartmentalize multiple ion channels involved in SAN pacemaker activity, such as canonical contributors to the SAN AP such as HCN channels (131), L-type calcium channels, and K_v_1.5 channels (132), as well as anion channels such as SWELL1 (LRRC8a), ClC-2, and ClC-3 (59, 103, 105, 108). Stretch-induced disruption of caveolae may participate directly or indirectly via localization of other signaling factors (133) in the activation of mechanoresponsive ion channels, including VRAC and ClC ion channels responsible for I_Cl,SWELL_ (134). Specific surface membrane proteins may not only affect changes in membrane potential but also directly or indirectly regulate intracellular Ca^2+^ cycling; on the other hand, intracellular Ca^2+^ cycling proteins may also regulate V_m_ via Ca^2+^ modulation of surface membrane electrogenic molecules (135).

## Mechanochemical Signal Transduction

**Figure 4:**
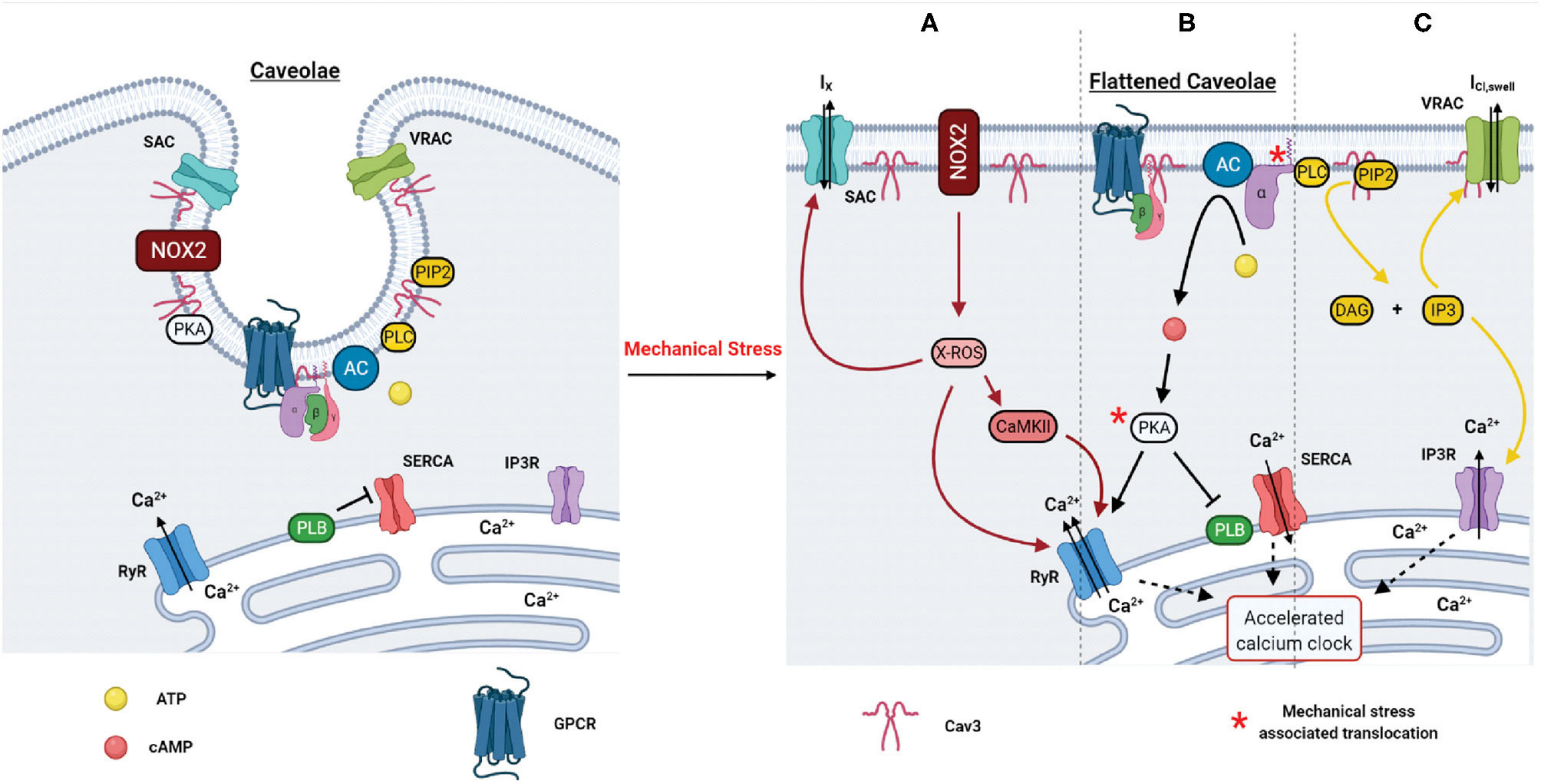
Proposed effect of mechanical loading on caveolae-associated signaling pathways and their effect on sinoatrial node calcium clock function. Mechanical stress: **(A)** Activates X-ROS signaling via NOX2 (NAPDH oxidase 2) which stimulates CaMKII activity and upregulates RyR (ryanodine receptor) function leading to an accelerated calcium clock, as well as sensitizes SAC (stretch-activated channels) for increased activation. **(B)** Displaces the GPCR alpha subunit, activating AC (adenylyl cyclase)-mediated conversion of ATP to cAMP, which activates PKA leading to an accelerated calcium clock via RyR activation and PLB (phospholamban) inhibition of SERCA (sarcoplasmic reticulum Ca^2+^-ATPase). **(C)** Induces PLC (phospholipase C)-mediated conversion of PIP2 to IP_3_ (inositol triphosphate), which activates IP_3_ receptors (IP_3_R) and an accelerated calcium clock and also activates VRACs (volume regulated anion channels). All three pathways lead to accelerated cardiomyocyte calcium cycling, LCR (local calcium releases) rate, and increased activity of mechanosensitive channels.

While changes in cardiac morphology are attributed to mechano-electrical signal transduction via regulating the activity of mechanosensitive ion channels, mechanochemical signal transduction could be described as a mechano-induced regulation of various second messenger signaling pathways that are ultimately translated into changes of calcium handling and ion channel activity. Here, we focus on mechano-dependent regulation of ROS, cAMP, and IP_3_ signaling pathways (Figure 4). It should be noted that mechano-electrical and mechanochemical feedbacks are not mutually independent but rather interact in a complex and dynamic manner as described below. While mechanochemical signal transduction pathways could be involved in the regulation of various ion channels via different post-translational modifications (such as phosphorylation, nitrosylation, and oxidation), activation of mechano-electrical feedback can significantly modify intracellular ion composition affecting intracellular Ca^2+^ signaling. Below, we briefly summarize several key mechano-chemical signaling pathways that could be involved in SAN mechanosensitivity. Though the role of these pathways has not been demonstrated in SAN mechanosensitivity, it is feasible that they have an impact on mechanical heart rate modulation.

Petroff et al. (33) were the first to use confocal microscopy to monitor subcellular Ca^2+^ events in cardiomyocytes during stretch and to provide direct evidence that stretch modulates the elementary Ca^2+^ release process, the Ca^2+^ spark. Stretch-induced increases in Ca^2+^ spark frequency are a phenomenon consistently observed in myocytes (136, 137), also in response to other mechanical stimuli, such as shear stress and afterload (123). A single myocyte stretch event immediately—within milliseconds—triggers a burst of Ca^2+^ sparks, which is reversible and declines within seconds (137, 138). While in ventricular myocytes these sparks are restricted in time and space, unique patterns of RyR expression and the presence of bridging RyR groups between large clusters demonstrated in the SAN cells (139–141) could lead to the generation of propagating LCR events as demonstrated in modeling studies by Stern and colleagues (141). This mechanism of stretch-induced increase in Ca^2+^ spark activity might be also present in the SAN and potentially contribute to mechanical regulation of Ca^2+^-clock activity (Figure 4).

### NOX2–Reactive Oxygen Species

Mechanical modulation of Ca^2+^ spark activity was linked to stretch-induced activation of ROS signaling that is also graded in a stretch-dependent manner (142). The stretch-induced NOX2-dependent ROS response sensitizes RyR to Ca^2+^ possibly through direct oxidation but may also do so indirectly via oxidation of calmodulin, displacing it from the RyR and promoting activation (143) or via RyR phosphorylation by oxidized CaMKII (144). These pathways of mechano-transduction are termed X-ROS signaling and require an intact microtubule network and functions independently of stretch-activated channels (SACs) and transsarcolemmal Ca^2+^ influx (33, 137). Furthermore, X-ROS signaling is confined to dyads (the cytosolic space between the sarcolemmal and SR membranes) (145) and has been proposed to be an important regulator of beat-to-beat adaptation to hemodynamic load in working cardiomyocytes (142). However, these pathways have not been confirmed specifically in SAN myocytes, but they are known to contain the necessary components (146).

In addition to regulation of Ca^2+^ signaling, the X-ROS pathway has also been found to be involved in the modulation of mechanoresponsive ion channels as well (147). Patch-clamp studies on stretched ventricular myocytes revealed NOX-dependent modulation of SACs (148), and this modulation may be facilitated by co-localization of NOX2 and SAC in caveolae (149, 150). Although SACs are not involved in X-ROS signaling per se, these channels may contribute to stretch-induced modulation of AP as discussed above for mechano-electrical signal transduction. Additionally, *I*_Cl,SWELL_ activated by osmotic swelling has been found to be controlled by an angiotensin II-dependent ROS cascade that is previously implicated by integrin stretch (113). This is consistent with persistent activation of *I*_Cl,SWELL_ and ROS present in several models of cardiac disease. Furthermore, Gradogna et al. demonstrated that LRRC8 channel subunits and their currents are differentially modulated by oxidation depending on LRRC8 channel subunit composition (151). Given that inflammation and oxidation are present in the setting of hypertension, it is possible that SAN mechanosensitivity could differ from other cardiac regions due to varying SWELL1 subunit expression (151).

### Nitric Oxide Synthase

Nitric oxide synthase (NOS) also plays a discrete role facilitating cardiac stretch as Petroff et al. demonstrated that stretch increases nitric oxide (NO) production with concurrent increases in Ca^2+^ spark frequency and transient amplitudes (33). Pharmacological inhibition or genetic deletion of both neuronal NOS (nNOS) and endothelial NOS (eNOS) demonstrates that subtypes contribute to the increase of systolic Ca^2+^, but only nNOS participated in the afterload induced Ca^2+^ sparks (152). Due to the short lifetime of NO, its effective signaling range is limited and dependent on the diffusion distance, amount produced, and the buffer capacity of the cell (33). Therefore, one possible explanation for the distinct effects of nNOS vs. eNOS-derived NO on Ca^2+^ sparks is their different subcellular localizations. While eNOS is localized at the caveolae (153, 154), nNOS is preferentially localized at the SR membrane in the vicinity of RyR, and nNOS increases RyR Ca^2+^ leak, directly by S-nitrosylation or indirectly via CaMKII (155). In addition, nNOS facilitates SERCA Ca^2+^ reuptake (155), which may compensate for the increased SR Ca^2+^ leak and reduced basal *I*_Ca,L_ (156). In regard to the SAN, in a study by Vila-Petroff et al. using exogenous NO donors, high levels of NO induced a large increase in cGMP and a negative inotropic effect, while low levels of NO increased cAMP and caused positive inotropy via cGMP-independent activation of adenylyl cyclase (157). Furthermore, it has been shown that inhibition of NOS has a negative chronotropic effect on SAN activity and that NOS activation has an opposite effect, indicating that SAN function is somewhat dependent on NOS activity (158, 159). However, unlike X-ROS signaling, NO mechanosensitivity operates on a slower time scale of minutes rather than seconds (33), suggesting that it may play a more significant role in conditions where chronic stretch is a factor (i.e., hypertension and chamber filling pressures).

### Atrial Natriuretic Peptide

Another important factor in myocyte stretch signaling is atrial natriuretic peptide (ANP) (160). Similar to X-ROS, ANP is a mechanosensitive signaling factor that is activated by a caveolae and angiotensin II-dependent pathway (161). ANP has been found to enhance reflex bradycardias (162); therefore, it is likely that ANP has a compensatory mechanosensitive role on the SAN, acting to restore it to normal function in response to elevated stretch. ANP has been found to shift midpoint activation of pacemaking *I*_f_ current toward less negative potentials (163) and thus accelerate SAN rhythm. ANP is also able to increase intracellular cGMP and cAMP levels (163), which play a crucial role in SAN automaticity via phosphorylation of the calcium clock proteins (50, 164). Indeed, ANP has been identified as a critical regulator of SAN automaticity (38, 165).

### Inositol Trisphosphate

IP_3_Rs are another type of SR Ca^2+^ release channels, which are activated by IP_3_ through the hydrolysis of phosphatidylinositol-(4, 5)-bisphosphate by phospholipase C and thus may also contribute to the LCR generation via hypersensitization of RyRs. They are highly abundant in atrial and SAN myocytes (166–168), and recent studies demonstrated that this signaling pathway might be confined within specific microdomains, including lipid rafts and dorsal ruffles (169). Stimulation of IP_3_Rs was found to accelerate spontaneous beating rate of the mouse SAN likely through regulation of Ca^2+^ spark activity and RyR function (170). In rabbit ventricular myocytes, upregulation of IP_3_R-induced Ca^2+^ releases was detected and linked to enhanced spontaneous SR Ca^2+^ releases (170). It has been shown that mechanical stretch can directly activate phospholipase C with production of IP_3_ (171), which may subsequently modulate SAN automaticity (Figure 4).

### Mechanochemical Signal Transduction and Caveolae

While there are numerous mechanochemical signaling factors that may affect SAN automaticity, they are united as facilitators of cardiac mechano-transduction through their association with caveolae membrane structures (Figure 4) (123, 172–174). For example, NOS (173), NOX2-mediated ROS (150), and calcium dynamics (123) are all affected by the presence of caveolae, which are suspected to play an inhibitory role on these factors, which are disrupted by shear stress. Digging deeper, angiotensin II mediates activation of cAMP production (175) and X-ROS through caveolae membrane structures (176), further linking the discussed factors to these structures. The suspected regulation of these signaling factors of SAN automaticity by caveolae may explain the connection between caveolae loss and cardiac pathology (177) as chronic shear stress depletes caveolae (178), allowing these factors to activate constitutively and/or enter unusual feedback loops. For example, as shear stress increases, caveolae flatten and release NOS, which should reduce the initial mechanical stimuli and allow caveolae to reform. However, if the mechanical stimulus is prolonged, membrane caveolae density will decrease, eliminating a crucial regulator of nNOS activity. Without this negative regulation, these signaling factors can enter positive feedback loops, inducing the generation of excess ROS from sarcolemmal and mitochondrial sources that can ultimately lead to changes in myocyte electrophysiology as calcium kinetics are subsequently altered. For these reasons, it is highly plausible that SAN caveolae may regulate downstream signaling factors that are known to alter SAN automaticity and consequently heart rate.

## Pathophysiology

While physiological stretch provides a critical autoregulatory feedback loop to adjust SAN pacemaker rate upon hemodynamic changes, pathophysiological stretch (and physiological stretch applied to diseased myocardium) can lead to SAN dysfunction and trigger cardiac arrhythmias (6, 7, 179). It has been shown that conditions associated with atrial pressure and/or volume preload/afterload, including heart failure, atrial fibrillation, hypertension, and valvular disease, are common comorbidities linked to SAN dysfunction or sick sinus syndrome (23, 180–183). Sick sinus syndrome is manifested clinically as arrhythmias that can include sinus bradycardia, sinus pauses or arrest, sinoatrial exit block, or alternating brady- and tachyarrhythmias (184). These manifestations can lead to chronotropic incompetence, which is an inadequate heart rate response to exercise or stress (184). Electrophysiological mechanisms that underlie SAN dysfunction in the setting of pathologically elevated atrial stretch are not completely understood and may vary for different conditions. The mechanisms could include abnormal functioning, expression, and/or regulation of the components of mechano-electrical and mechanochemical signal transduction and may be also conditioned by structural remodeling of the SAN.

Importantly, in the setting of sinus node dysfunction when SAN is not able to maintain physiologically robust rhythm, mechanical stretch can enhance automaticity in latent atrial pacemakers or provoke arrhythmogenic activity in the working myocardium to form ectopic foci and trigger atrial fibrillation (30, 185, 186). Though distribution patterns of stretch-induced atrial ectopic foci are not currently known, pulmonary veins represent the most common source of atrial fibrillation ectopy (187, 188). Mechanical stretch of pulmonary vein myocardium has been shown to promote arrhythmogenic activity from this region and may initiate atrial fibrillation (186, 189). Recently, we have demonstrated that stretch-induced activation of *I*_Cl,SWELL_ in rat pulmonary veins leads to membrane depolarization and decreased AP amplitude, which trigger conduction discontinuities and both ectopic and reentrant activities (30, 59). We also found that downregulation of caveolin-3 protein expression and disruption of caveolae structures during chronic hypertension in spontaneously hypertensive rats significantly facilitates activation of *I*_Cl,SWELL_ and increase the sensitivity of pulmonary vein in response to stretch to 10- to 50-fold (59). The increased sensitivity to stretch could be linked to the presence of constitutively active *I*_Cl,SWELL_ that has been previously reported in failing (a pacing-induced congestive heart failure model) canine ventricular myocytes (111) and in human atrial myocytes obtained from patients with right atrial enlargement and/or elevated left ventricular end-diastolic pressure (112). Similar results of constitutively (i.e., without hypotonic stress) active, DIDS-sensitive *I*_Cl,SWELL_ current was shown in cultured neonatal rat ventricular hypertrophic myocytes induced by cyclic mechanical stretch (190) and in mouse ventricular myocytes isolated from hearts subjected to 4 weeks' transverse aortic constriction (TAC) (191).

Pressure overload for 8 weeks using the TAC mouse model demonstrated a smaller basally active *I*_Cl,SWELL_ as well as a significantly reduced hypotonic solution-induced *I*_Cl,SWELL_ (191). Similar decreases in hypotonic *I*_Cl,SWELL_ current without basal activation are observed in rabbit hypertrophied ventricular cells after treatment of volume and pressure overload (192) and spontaneous hypertrophic ventricular cells from caveolin-3-deficient mice (193). These may indicate that in an early adaptive stage of cardiac pressure/volume overload, *I*_Cl,SWELL_ is basally activated by persistent mechanical stretch of the cell membrane and thus contributes to SAN tachycardia as well as facilitate atrial ectopy, as discussed earlier. However, attenuation of *I*_Cl,SWELL_ mechanical sensitivity by long-term mechanical stretch of the plasma membrane may contribute to depressed SAN function and contribute to transformation to a non-adaptive stage. Indeed, our preliminary findings indicate that in 8-week post-myocardial infarction mouse model of heart failure, mRNA protein expression levels of ClC-2 and ClC-3 mechanosensitive chloride channels are significantly downregulated within the intercaval region of the right atrium, which correlates with a significantly enhanced cardiomyocyte membrane tension and downregulation of caveolae structures (194).

Pathological stretch can also affect mechanochemical signal transduction and contribute to stretch-mediated ectopic foci and atrial arrhythmogenesis. Stretch-induced activation of ROS systems via activation and upregulation of NADPH oxidases NOX2 and NOX4 have been linked to an increase in oxidation of RyRs and concomitant rise in spontaneous Ca^2+^ release event frequency, elevated Ca^2+^ leak, and significant increase in atrial fibrillation susceptibility (195). It should be also noted that chronic mechanical stretch may dramatically attenuate the protein expression profile of various ion channels and Ca^2+^-handling proteins, including those involved in mechano-electrical and mechanochemical signal transductions, further contributing to SAN dysfunction and atrial arrhythmogenesis.

## Summary

Emerging evidence demonstrates that mechano-electrical and mechanochemical signal transduction pathways could be implicated in mechanical modulation of SAN function and thus represent an important mechanism for intrinsic regulation of cardiac rhythm. This adds another level of complexity to SAN automaticity and could be described as a “mechanics-clock” component of the pacemaker system (6, 7). Though the exact components of mechano-electro-chemical signal transduction involved in SAN mechanosensitivity are currently unknown, as summarized in the current review, these may involve a number of complex signaling feedback mechanisms that alter the function of both the voltage and calcium pacemaker clocks. As discussed, these mechanisms may interplay with each other, providing a precise attenuation of the SAN beating rate in response to various mechanical stimuli. Disruption of SAN function and regulation has been observed with multiple pathological conditions that are associated with atrial pressure/volume overload and thus may involve the remodeling of the components of the mechano-electro-chemical feedback loops in the SAN. Identification of such components, their impact into SAN pacemaking, and pathological remodeling may provide new therapeutic targets for the treatment of SAN dysfunction and associated rhythm abnormalities. Moreover, linking molecular components of mechano-electro-chemical signaling to certain cellular nanodomains and nanostructures may introduce a novel framework for therapeutic approaches for pacemaker dysfunction treatment targeted at preventing the degradation of cardiac cytoarchitecture.

## Author Contributions

RS and AG conceived the topic of the review and steered the general direction on what they wanted the review to cover. DT, CK, and JH examined the details of SAN mechanosensitivity and organized the literature into a concise review. PM and MM produced, analyzed, and wrote significant portions of the review related to RNAseq data. All authors allocated time and effort into writing/editing the manuscript and creating figures, small differences in effort are reflected in the author order.

## Conflict of Interest

The authors declare that the research was conducted in the absence of any commercial or financial relationships that could be construed as a potential conflict of interest.

## Publisher's Note

All claims expressed in this article are solely those of the authors and do not necessarily represent those of their affiliated organizations, or those of the publisher, the editors and the reviewers. Any product that may be evaluated in this article, or claim that may be made by its manufacturer, is not guaranteed or endorsed by the publisher.
