# Supplementary material for: Electrophysiological and Molecular Mechanisms of Sinoatrial Node Mechanosensitivity
Source: Front Cardiovasc Med. 2021 Aug 9;8:662410. doi: 10.3389/fcvm.2021.662410 (PMC8382116; doi:10.3389/fcvm.2021.662410)
Supplement: Supplementary file 1 [file Data_Sheet_1.PDF]

Supplementary Materials

Methods

Mouse SAN RNA Sequencing:

Poly-A based mRNA enrichment and reverse transcription: RNA-Seq libraries were constructed with the Truseq stranded mRNA sample preparation (Low throughput protocol) kit from Illumina. Five hundred nanogram of total RNA was used for the construction of the libraries. The first step in the workflow involves purifying the poly-A containing mRNA molecules using poly-T oligo attached magnetic beads. Following purification, the mRNA is fragmented into small pieces using divalent cations under elevated temperature. The cleaved RNA fragments are copied into first strand cDNA using SuperScript II reverse transcriptase, Actinomycin D and random hexamer primers. The Second strand cDNA was synthesized by replacing dTTP with dUTP. These cDNA fragments then have the addition of a single 'A' base and subsequent ligation of the adapter. The products are then purifiedand enriched with 15 cycles of PCR. The final cDNA libraries were validated with a Fragment Analyzer system (Advanced Analytical Technologies, Ankeny, IA) and quantified with a KAPA qPCR kit.

Sequencing: For each sequencing lane of a flowcell V4, three libraries were pooled in equal proportions, denatured with NaOH and diluted to 22 pM before clustering. Cluster formation, primer hybridisation and single end-read 50 cycles sequencing were performed on cBot and HiSeq2500 (Illumina, San Diego, CA) respectively.

Sequencing quality control: Image analyses and base calling were performed using the Illumina HiSeq Control Software and the Real-Time Analysis component. Demultiplexing was performed using Illumina’s conversion software (bcl2fastq 2.20). The quality of the raw data was assessed using FastQC from the Babraham Institute and the Illumina software SAV (Sequencing Analysis Viewer). Potential contaminants were monitored with the FastQ Screen software from the Babraham Institute.

RNA-Seq data analysis: We aligned RNA-seq reads to the mouse genome (UCSC mm10) with the splice junction mapper TopHat 2.1.11, which used Bowtie 2.3.4.32. We

downloaded gene model annotations from the UCSC database (genes.gtf 15 January 2019). Final read alignments having more than 3 mismatches were discarded. We performed gene counting using featureCounts 1.6.23. Counts were normalized using the trimmed mean of M-values (TMM) method implemented in the Bioconductor4 package edgeR 3.20.15.
